# Application of percutaneous lymphatic contrast-enhanced ultrasound in lymphovenous anastomosis microsurgery

**DOI:** 10.1371/journal.pone.0330773

**Published:** 2025-08-22

**Authors:** Jing Wang, Ming Xing Hu, Min Lu, Xu Li

**Affiliations:** 1 Department of Ultrasound Medicine, Bishan Hospital of Chongqing Medical University, Bishan Hospital of Chongqing, Chongqing, China; 2 Lymphatic Microsurgery, Bishan Hospital of Chongqing Medical University, Bishan Hospital of Chongqing, Chongqing, China; University of Ghana Noguchi Memorial Institute for Medical Research, GHANA

## Abstract

**Objective:**

This study evaluated percutaneous lymphatic contrast-enhanced ultrasound (CEUS) for preoperative localization in preparation for lymphovenous anastomosis (LVA) microsurgery.

**Methods:**

Fourteen healthy volunteers and 14 patients with lower limb lymphoedema were studied. SonoVue® (Bracco, Milan, Italy) was used to measure lymphatic vessel diameters and depths in the dorsal foot, ankle, and lower leg of the subjects. In the lymphoedema patients, lymphatic vessels were observed for continuity, distortion, dilation, interruption, and other abnormalities. On the basis of the CEUS images, the lower limb lymphatic vessels were categorized as normal, dilated, contracted, or sclerotic. In the lymphoedema patients, the locations of lymphatic vessels with good visibility were marked on the skin, and the accuracy of preoperative localization was evaluated based on the basis of surgical results. The data were analysed using IBM SPSS Statistics27.0 (IBM Corp., Armonk, NY, USA). Continuous data are expressed as the means ± standard deviations and were compared using paired t-tests, with *P* < 0.05 considered statistically significant.

**Results:**

Among the healthy volunteers, one had a 1–2 mm lymphatic vessel visible in the dorsal foot, whereas 13 had no lymphatic vessels visible in the dorsal foot; however, lymphatic vessels were visible in the ankle and lower leg in all volunteers, with an average diameter of 0.42 ± 0.09 mm, resulting in a 100% visualization success rate. In lymphoedema patients, CEUS achieved a 92.86% success rate (13/14) in visualizing lymphatic vessels within 1 minute in lymphedema patients, excluding one patient with primary lymphoedema; the average vessel diameter was 0.66 ± 0.24 mm. The most common type consisted of dilated lymphatic vessels with tortuous morphology and increased diameter, often accompanied by reflux and interstitial dispersion in lymphoedema patients. With the successful intraoperative identification of lymphatic vessels under the surface marking during LVA as the standard, the accuracy of preoperative lymphatic vessel localization by CEUS was 92.36%.

**Conclusion:**

CEUS can accurately localize functional lymphatic vessels and serves as a valuable complementary method to indocyanine green for preoperative lymphatic vessel mapping in LVA.

## Introduction

Lymphedema is a condition caused by lymphatic obstruction or dysfunction, leading to the accumulation of lymph fluid in subcutaneous tissues. This condition is manifested as limb swelling, pain, or functional impairment. Lymphovenous anastomosis (LVA) is a significant treatment method for lymphoedema, making accurate preoperative localization of lymphatic vessels crucial [1–5]. Current preoperative localization techniques include MRI, radionuclide imaging, and fluorescence imaging [6]. While MRI provides high-resolution images and clear tissue information, its high cost and contraindications limit its use [7]. Radionuclide imaging offers real-time lymphatic flow information but poses radiation exposure risks. Fluorescence imaging is capable of real-time monitoring of fluorescent signals. Indocyanine green (ICG) has the advantages of real-time results and repeatability, which make it useful for real-time intraoperative identification of lymphatic vessels during operation and immediate postoperative evaluation of anastomosis drainage. This method is the reference standard for lymphatic system imaging in LVA [8]. However, its maximum penetration depth is limited to 1–2 cm beneath the skin surface. Moreover, in patients with cutaneous lymphatic reflux, the accumulation of large amounts of fluorescent contrast agent in the skin can obscure the visualization of deeper lymphatic vessels [9]. Therefore, each of these methods has limited use. High-frequency ultrasound is economical, convenient, and radiation-free, and there has been progress in its application to lymphatic imaging in recent years [10–15]. However, the average depth of the lymphatic vessels is approximately 5.2 mm. In cases of lymphoedema, conventional high-frequency probes struggle to distinguish between lymphatic vessels within the fascial layer and subcutaneous fluid accumulation caused by lymph leakage, The visibility of lymphatic vessels is compromised, making it difficult to meet the clinical requirements for clear visualization [10]. CEUS is a well-characterized effective technique for evaluating sentinel lymph node metastasis in patients with breast cancer, allowing real-time visualization of both lymphatic vessels and sentinel lymph node morphology [16]. Given its established value in sentinel lymph node assessment, CEUS may also offer important advantages in preoperative planning for lymphatic surgeries. However, studies on its use in LVA preoperative localization are limited. This study aimed to verify the feasibility of CEUS as an effective imaging technique for accurately locating lymphatic vessels preoperatively.

## Materials and methods

### Study subjects

**Fig 1:**
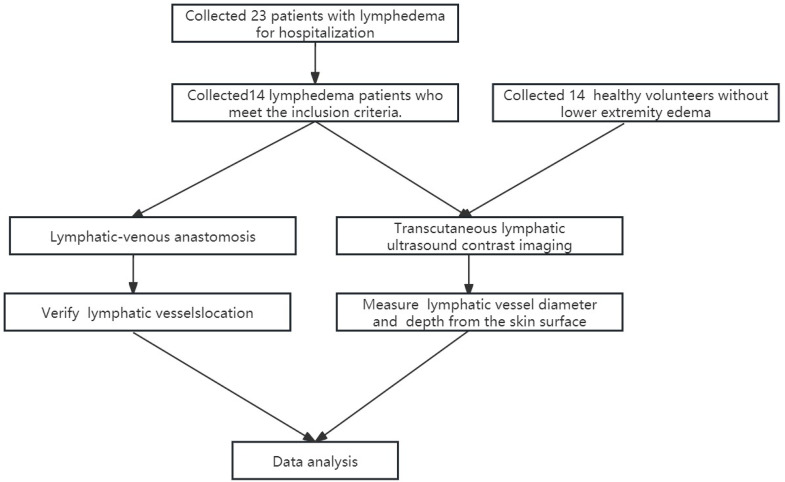
Flowchart of patient selection.

For this retrospective study, fourteen patients with lower limb lymphoedema in the Department of Lymphology, Bishan Hospital affiliated with Chongqing Medical University, who were admitted between 01/05/2023 and 31/05/2024, were selected. The data were accessed retrospectively between 01/12/2024 and 01/02/2025.Patients were eligible for inclusion if they underwent CEUS examination, had a clinical diagnosis of lower limb lymphodema, and were scheduled to undergo LVA surgery. In addition, a group of 14 healthy volunteers without lower limb lymphedema was recruited to establish the normal reference range for lymphatic vessel diameter. The inclusion criteria for healthy volunteers were as follows: they had undergone CEUS examination, had no signs or history of lower limb lymphedema, and had no prior history of varicose veins or lower limb surgery. The exclusion criteria for all participants included the presence of severe hepatic or renal dysfunction, respiratory failure, heart failure, active immune system diseases, or autoimmune disorders. Individuals with known allergies to contrast agents were also excluded from the study, as were pregnant or lactating women,. A total of 105 lymphatic vessels in lymphoedema patients and 46 lymphatic vessels in healthy volunteers met the inclusion criteria. A flowchart of the selection process for this study is shown in Fig 1.

### Contrast-enhanced ultrasound

This study was approved by the Medical Ethics Committee of Bishan Hospital of Chongqing Medical University and conducted in accordance with the Declaration of Helsinki (1964) and its later amendments. The ethics approval ID is Scientific Ethics Review cqbskyll-20241128–03, and the approval date was 29 November 2024. All data were fully anonymized before analysis. Written informed consent was obtained from all participants for the use of their medical records.

#### Instruments.

A Philips EPIQ 5 Color Doppler ultrasound system (Philips Healthcare, Bothell, WA, USA) with a high-frequency linear probe (4–18 MHz) was used for all examinations. The contrast agent used was SonoVue® (Bracco, Milan, Italy), which consists of 25 mg of lyophilized powder containing sulfur hexafluoride (SF₆) microbubbles (59 mg), reconstituted with 5 mL of normal saline to form a homogeneous suspension.

#### Procedures.

Subjects lay supine with the lower limbs and feet exposed. A coupling agent was applied, and the probe was held vertically without pressure to avoid compressing the lymphatic vessels. After the first and second toe gaps and the lateral and medial ankles were disinfected, 0.5 ml of the contrast agent suspension was injected with a 21G needle. Superficial organ contrast mode was activated, the depth was adjusted to 2.5 cm, and low-mechanical-index imaging (MI = 0.06) with the focus in the far field was used to reduce microbubble destruction. The dynamic range was adjusted to a maximum of 72 MHz to minimize tissue signal suppression. After injection, the site was gently massaged with a cotton swab, and the lymphatic vessels in the dorsal foot, ankle, and lower leg fascial layers were sequentially located. The probe was used to track lymphatic vessels along their long axis, observing the continuity of the imaging, noting any distortion, dilation, interruption, non-visualization, lymphatic cysts, or collateral openings, as well as any reflux of the contrast agent within the lymphatic vessels or from the lymphatic vessels to the skin. Measurements of the diameter of the lymphatic vessels and their depth under the skin were recorded. Lymphatic vessels with good visualization in lymphoedema patients were marked on the skin.

### Statistical analysis

IBM SPSS Statistics27.0 (IBM Corp., Armonk, NY, USA) was used for data analysis. Normality tests were conducted for continuous data. Data conforming to a normal distribution are expressed as the mean ± standard deviation and were compared using paired t-tests. Categorical data were compared using chi-square tests. Non-normally distributed data are expressed as the median ± interquartile range and were compared using nonparametric rank-sum tests. A P-value <0.05 was considered to indicate statistical significance.

## Results

### General conditions

A total of 14 patients with lower limb lymphoedema and 14 healthy volunteers participated in this study. The detailed demographic and clinical characteristics of the two groups are summarized in Table 1 and Table 2, respectively. There were substantial differences between the patient group and the healthy volunteers group in terms of age, body mass index, and comorbidities. Therefore, no direct comparisons were conducted between the two groups.

**Table 1:** General conditions of patients with lower limb lymphedema.

Age (years), mean ± SD (range)	59.21 ± 2.20 (44-74)
Sex (male/female)	2/12
Diabetes mellitus, n (%)	4/10 (28.57%)
Hypertension, n (%)	10/4 (71.43%)
BMI (kg/m²), mean ± SD (range)	26.09 ± 0.81 (18.8-30.8)
Duration of Lymphoedema (years), mean ± SD (range)	6.42 ± 1.06 (1234567891011–12)
Initial site of edema, n	Perineums (2),Lower limbs (12)
Cause of lymphoedema, n	Primary:1
	Secondary: 13 (thoracic duct stenosis:1; endometrial cancer:4; ovarian cancer:3;,cervical cancer:5)

**Table 2:** General conditions of healthy volunteers.

Age (years), mean ± SD (range)	37.36 ± 9.73 (22-52)
Sex (male/female)	3/11
Diabetes mellitus, n (%)	1/13 (7.14%)
Hypertension, n (%)	0/14 (0%)
BMI(kg/m²), mean ± SD (range)	22.21 ± 4.28 (16.6-32.5)

### CEUS images from patients with lower limb lymphoedema

**Fig 2:**
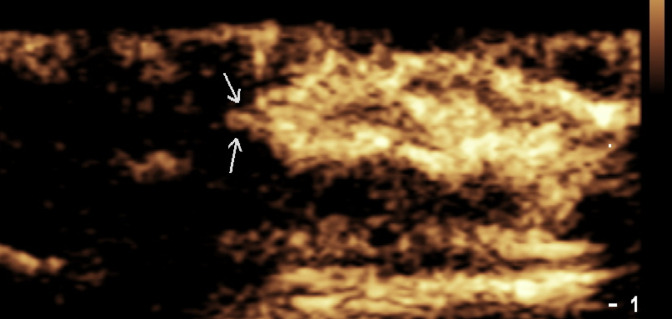
Contrast-enhanced ultrasound image of lower limb lymphatic vessels with minimal contrast agent uptake in a patient with lymphedema; the arrows indicate the lymphatic vessel.

In 14 lower limb lymphoedema patients, lymphatic vessels were visualized in all but one primary lymphoedema patient. A total of 105 lymphatic vessels were localized preoperatively (41 in the dorsal foot, 43 in the ankle, and 21 in the lower leg), with a CEUS success rate of 92.86%. The average diameter of the lymphatic vessels was 0.66 ± 0.24 mm, and the average depth under the skin was 5.46 ± 2.21 mm. Thirteen patients showed clear lymphatic vessel enhancement within one minute after contrast agent injection. However, in three patients, the visualization was poor, with only faint lymphatic vessels observed at the margins of the injection site. Notably, these patients had worn compression stockings or bandages prior to the examination, which may have affected their lymphatic flow and contrast uptake. As illustrated in Fig 2, the lower limb lymphatic vessels in a patient with lymphoedema showed minimal uptake of the contrast agent, and contrast agent accumulation was limited to the periphery of the injection site.

### CEUS images from healthy volunteers

Among the 14 healthy volunteers, one subject had a 1–2 mm lymphatic vessel visible in the first toe gap after contrast agent injection, whereas the remaining 13 had no lymphatic vessels visible in the dorsal foot. All 14 volunteers had visible lymphatic vessels in the ankle and lower leg segments within 1 minute, with a total of 46 visualized lymphatic vessels. The average diameter was 0.42 ± 0.09 mm, and the average depth under the skin was 6.16 ± 3.80 mm.

### Comparison of lower limb lymphatic vessels between healthy volunteers and lymphoedema patients

Lymphatic vessel diameters were significantly greater in patients with lymphoedema than in healthy volunteers (*P* < 0.05), suggesting pathological dilation of lymphatic vessels in the disease group. This finding is summarized in Table 3. In contrast, no significant difference was found in the depth of the lymphatic vessels under the skin surface between the two groups.

**Table 3:** Comparison of lower limb lymphatic vessels between healthy volunteers and lymphoedema patients.

Group	Healthy volunteers	Lymphoedema patients	p	t
Diameter	0.42 ± 0.09	0.66 ± 0.24	0.001	−3.845
Depth from skin	6.16 ± 3.80	5.46 ± 2.21	0.998	0.003

### Classification of lower limb lymphatic vessels in lymphoedema patients based on preoperative CEUS

**Fig 3:**
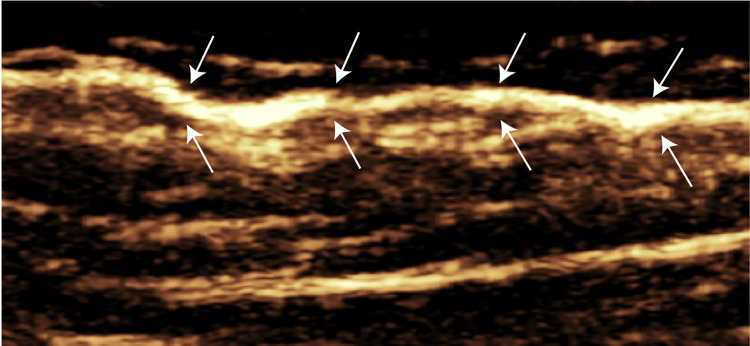
Contrast-enhanced ultrasound image from a healthy volunteer. AArrowss indicate the lymphatic vessel.

**Fig 4:**
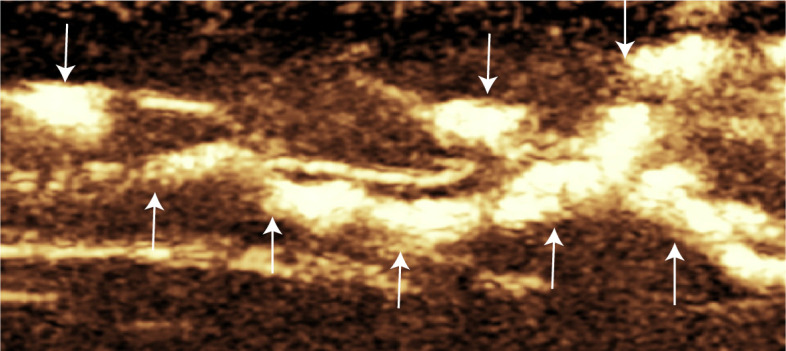
Contrast-enhanced ultrasound image of dilated-type lower limb lymphatic vessels in a patient with lymphedema. Arrows indicate lymphatic vessel.

Lymphatic vessel degeneration can be classified pathologically into four types: normal, dilated, constricted, and sclerotic [17]. In this study, lower limb lymphatic vessels were categorized into these four types on the basis of CEUS findings as summarized in Table 4. Normal-type vessels appeared as smooth, linear structures with continuous contrast enhancement and no signs of intralymphatic or dermal backflow. Their diameters were ≤ 0.7 mm, which is consistent with the upper limit observed in healthy volunteers. An example of a normal-type lymphatic vessel is shown in Fig 3. Dilated-type vessels, the most common subtype observed, presented diameters > 0.8 mm or, in some cases, diameters ≤ 0.7 mm with evidence of intralymphatic or dermal reflux. These vessels often displayed a tortuous or irregular course. A representative dilated lymphatic vessel, as shown in Fig 4, demonstrates both contrast enhancement and vessel tortuosity, suggesting compensatory remodelling. Constricted-type vessels were characterized by discontinuous, faint enhancement and an irregular, narrowed course. This pattern is exemplified in S1 Video, which shows segmental uptake and poor contrast flow. Sclerotic-type vessels showed no enhancement beyond the injection site, indicating complete obstruction or fibrosis of the lymphatic channel. Additionally, Intraluminal and cutaneous reflux were more commonly observed in dilated-type lymphatic vessels. Representative cases are shown in S2 Video (intraluminal reflux) and S3 Video (cutaneous reflux).

**Table 4:** Preoperative classification of lower limb lymphatic vessels in lymphoedema patients using contrast-enhanced ultrasound.

Type	Number	Intraluminal reflux	Cutaneous reflux	Interstitial diffusion
Normal type	26	0	0	0
Dilated type	55	46	6	5
Constricted type	24	5	0	1
Sclerotic type	0	0	0	0

### Preoperative-intraoperative comparison of lymphatic vessels in patients with lymphedema

**Fig 5:**
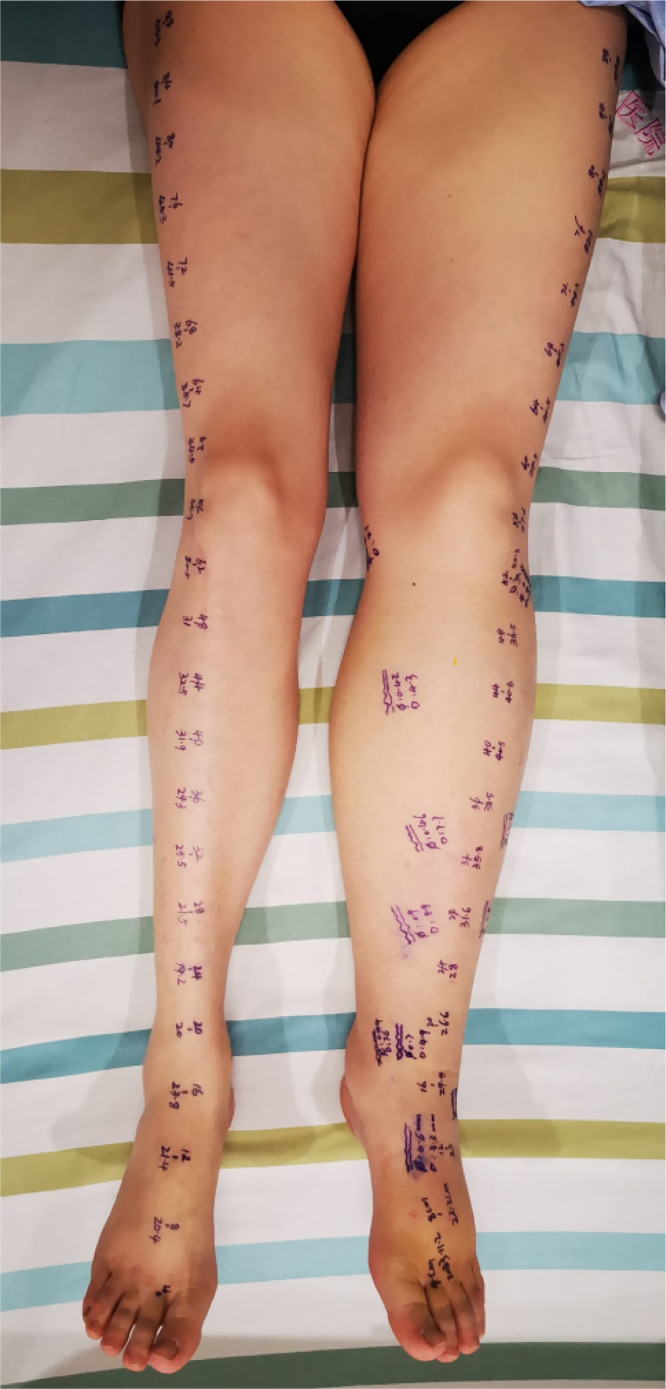
Preoperative Line marking of the lymphatic vessels.

Preoperative skin markings were made over the lymphatic vessels to indicate that the lymphatic vessels were located in the fascial layer at the marked sites; these markings were confirmed intraoperatively by surgeons as shown in Fig 5, demonstrating that CEUS achieved a localization accuracy of 92.36%.

## Discussion

Lymphoedema is a chronic disease classified into primary and secondary types. Secondary lymphoedema is caused mainly by factors such as surgery, trauma, or infection, which impede lymphatic return, leading to fluid accumulation in the superficial soft tissues of the limbs. Eventually, this accumulation of fluid results in fibrosis, fat sclerosis, fascia thickening, and overall limb hypertrophy [18].

Our study revealed that the average depth of lymphatic vessels in the lower limbs of lymphoedema patients was 5.46 ± 2.21 mm, which is comparable to the value of 5.2 mm reported by Hisako Hara et al. [1] However, the frequency range of conventional linear probes (9–18 MHz) provides poor resolution for tissues within 1 cm of the skin, making it difficult to clearly visualize the lymphatic vessel diameter and depth. Additionally, significant subcutaneous soft tissue oedema, increased fat echo, and subcutaneous fluid accumulation in lymphoedema patients reduce the clarity of greyscale ultrasound imaging. The lymphatic system returns excess fluid from the interstitial space to the bloodstream. The thoracic duct drains into the internal jugular vein, forming the anatomical basis for LVA, a potential treatment for lymphoedema [19]. CEUS has shown advantages in evaluating sentinel lymph node metastasis in patients with breast cancer by providing real-time visualization of lymphatic vessels and nodes [20,21]. These findings support its further application in LVA.

In this study, the accuracy of preoperative localization using percutaneous ultrasound lymphangiography was 92.36%, which was significantly greater than that of conventional ultrasound. This finding suggests that percutaneous ultrasound lymphangiography has distinct advantages for LVA preoperative localization, which is consistent with findings by Jia Zhu et al [22]. Our results indicate that the average diameter of lymphatic vessels in lymphoedema patients is 0.66 ± 0.24 mm, which is similar to the value of 0.65 ± 0.35 mm reported by Hara et al. [12] but different from the value of 0.33 mm reported by Hisako et al.[1] and the value of 1.2 ± 0.47 mm reported by Manon et al [23]. These differences may be due to varying stages of lymphoedema among the study subjects, affecting the degree of lymphatic vessel dilation.

Hayashi et al. [24] studied the lower limb lymphatic vessels of 26 healthy volunteers, reporting a diameter range of 0.2−.8 mm. Hisako Hara et al. [25] reported average diameters of 0.160 mm, 0.163 mm, and 0.164 mm in the supine, sitting, and standing positions, respectively. In our study, the diameter range for healthy individuals was 0.27–0.66 mm. These differences can be attributed to factors such as patient position, measurement technique (conventional versus contrast-enhanced ultrasound), examination site (dorsum of the foot versus calf or ankle), and patient characteristics (ethnicity, height, and weight). The variations in lymphatic vessel diameter observed among healthy subjects may be attributed to several factors. These include differences in the regions examined, as our study focused on the dorsal foot, ankle, and calf, while Hayashi et al. assessed the entire lower limb, and Hisako et al. targeted areas 20 cm above and 10 cm below the knee. In addition, variations in ethnicity, body habitus, and patient characteristics may have influenced the results. Discrepancies in measurement techniques also play an important role, as our study utilized contrast-enhanced ultrasound, whereas the other studies relied on conventional ultrasound, which may have inadvertently measured fascia or small veins instead of lymphatic vessels, leading to potential measurement errors.

Lahtinen et al. [26] studied 60 upper limb lymphatic vessels in 30 volunteers, and visualized 59 vessels as far as the mid-elbow within 18 seconds. In our study, all 14 healthy volunteers had visible lymphatic vessels in the ankle and calf, but only one case had a 1–2 mm vessel visible in the dorsal foot. In contrast-enhanced ultrasound, both enhanced lymphatic vessels within the adipose layer and fibrous septa appear as hyperechoic structures. In healthy individuals, the subcutaneous soft tissue on the dorsum of the foot is relatively thin, and the lymphatic vessels lie close to the hyperechoic fascial structures, making it difficult to distinguish small lymphatic vessels. This may be one of the reasons why lymphatic vessels on the dorsum of the foot were not visualized in the other 13 patients. Additionally, the small diameter of these vessels and the rapid lymphatic fluid flow within them may prevent their visualization. Current studies on lower limb lymphatic vessels using percutaneous ultrasound contrast are limited, necessitating further clinical research.

Normal lymphatic vessels demonstrate continuous contrast enhancement and unidirectional flow due to intact valve function. Dilated lymphatic vessels have increased intraluminal pressure, flattened endothelial cells, widened lumens, and some valve reflux, appearing as continuously enhanced, widened vessels with possible reflux on contrast imaging. Constricted lymphatic vessels, characterized by smooth muscle cell transformation into synthetic cells and by collagen fiber growth, show intermittent faint enhancement and tortuous paths. Sclerotic lymphatic vessels, which are primarily composed of fibrous elements, lose their transport and contractile abilities, leading to complete lumen obstruction. Sclerotic lymphatic vessels do not show contrast enhancement except at the subcutaneous injection site [27,28]. Therefore, percutaneous ultrasound contrast classification can indicate the pathological degeneration of lymphatic vessels. In this study, the detection rate of dilated lymphatic vessels was significantly greater than that of constricted and sclerotic vessels, which may be attributable to several factors. First, dilated lymphatic vessels have a patent structure with a superior capacity to retain and transport contrast agents, resulting in continuous and clear enhancement on CEUS. This feature also explains the high incidence of intraluminal reflux observed in this group (46 out of 55 cases). Moreover, dilated lymphatic vessels exhibit an increased diameter and often have a tortuous course, making them easier to identify via ultrasound. In contrast, constricted lymphatic vessels typically show segmental narrowing and impaired flow, leading to discontinuous and weaker contrast enhancement. Although constricted vessels displayed intraluminal reflux in some cases (5 out of 24 cases), insufficient contrast filling and irregular morphology make them more difficult to distinguish on imaging. Additionally, the smaller diameter of these vessels, especially in deeper tissues, increases the likelihood of confusion with surrounding structures, further complicating their identification.

Sclerotic lymphatic vessels are usually non-functional, with obliterated or highly fibrotic lumens that prevent contrast agent entry or flow. As a result, they rarely show enhancement on CEUS and may even be mistaken for surrounding fibrotic tissue. No clear sclerotic vessels were visualized in this study. Furthermore, the disease stage distribution among the enrolled patients may have influenced the detection rates of different vessel types. Most patients were in the early stages of lymphoedema, where dilated and constricted vessels predominate, whereas sclerotic vessels, which are more common in advanced stages, may have been underrepresented, introducing potential selection bias that would affect the detection results.

Finally, the technical limitations of CEUS itself may also contribute, for example, the increased acoustic attenuation in fibrotic or thickened subcutaneous tissues, further reduces the visibility of constricted and sclerotic lymphatic vessels. Mihara et al. [29] reported similar findings, with more non-sclerotic than sclerotic vessels in patients with lower limb lymphoedema. However, in the presence of dermal backflow or when lymphatic vessels are situated at greater depths, fluorescence imaging is unable to reliably detect intraluminal reflux in real time. In the study, 48.57% of vessels presented with intraluminal reflux preoperatively on CEUS, indicating valve dysfunction and providing valuable imaging evidence for surgical planning and prognosis.All preoperatively localized lymphatic vessels were anastomosed during surgery, with diameters ranging from 0.26 to 3.22 mm. Shen Yang [30] reported that LVA outcomes were better when lymphatic vessel diameters were ≥0.5 mm, a hypothesis we plan to test in future studies.

Although CEUS has demonstrated significant clinical value in assessing lymphatic vessel structure and dynamics, it has certain limitations, such as limited temporal and spatial resolution, tissue artefact interference,microbubble disruption, and variability due to vessel pathology and anatomical location, all of which affect image clarity. Multiple contrast agent injections and high-resolution linear probes are necessary for satisfactory ultrasound images. Additionally, common treatments for lymphoedema, such as compression therapy, may affect lymphatic vessel morphology and visualisability, suggesting that the examination timing should be adjusted accordingly.

The limitations of this study include potential selection bias, as all participants had lymphatic vessels visualized in the lower limbs. In addition, the classification of pathological lymphatic vessel types was based primarily on observations from a small number of volunteers, which may limit the generalizability of the results. Moreover, as this investigation was a retrospective single-center study, its findings require further verification through prospective multicentre trials conducted across different regions to fully assess the clinical value of contrast-enhanced ultrasound in LVA.

## Conclusion

CEUS is a promising non-invasive imaging technique with considerable value as a supplement to ICG lymphography for the preoperative localization of functional lymphatic vessels. In patients with dermal lymphatic reflux, where ICG often fails to visualize target vessels, CEUS can still effectively detect them. Its greater imaging depth also allows the identification of deeper lymphatic vessels, aiding in surgical planning. Although CEUS has clear advantages, larger and multicentre studies are needed to further validate and optimize its clinical use.

## Supporting information

S1 Dataset Statistical dataset of lymphatic vessel characteristics in healthy volunteers. (XLSX)

S2 Dataset Statistical dataset of lymphatic vessel characteristics in patients with lower limb lymphedema. (XLSX)

S1 Video Contrast-enhanced ultrasound image of constricted-type lower limb lymphatic vessels in a patient with lymphedema. (MP4)

S2 Video Contrast-enhanced ultrasound image showing intraductal reflux in lower limb lymphatic vessels in a patient with lymphedema. (MP4)

S3 Video Contrast-enhanced ultrasound image showing cutaneous reflux in lower limb lymphatic vessels in a patient with lymphedema. (MP4)
